# Conscious Sedation Procedures Using Intravenous Midazolam for Dental Care in Patients with Different Cognitive Profiles: A Prospective Study of Effectiveness and Safety

**DOI:** 10.1371/journal.pone.0071240

**Published:** 2013-08-05

**Authors:** Valérie Collado, Denise Faulks, Emmanuel Nicolas, Martine Hennequin

**Affiliations:** 1 Clermont Université, Université d’Auvergne, EA4847, Centre de Recherche en Odontologie Clinique, BP 10448, F-63000 Clermont-Ferrand, France; 2 CHU Clermont-Ferrand, Service d’Odontologie, Hôtel-Dieu, F-63000 Clermont-Ferrand, France; University of Toronto, Canada

## Abstract

The use of midazolam for dental care in patients with intellectual disability is poorly documented. This study aimed to evaluate the effectiveness and safety of conscious sedation procedures using intravenous midazolam in adults and children with intellectual disability (ID) compared to dentally anxious patients (DA). Ninety-eight patients with ID and 44 patients with DA programmed for intravenous midazolam participated in the study over 187 and 133 sessions, respectively. Evaluation criteria were success of dental treatment, cooperation level (modified Venham scale), and occurrence of adverse effects. The mean intravenous dose administered was 8.8±4.9 mg and 9.8±4.1 mg in ID and DA sessions respectively (t-test, NS). 50% N_2_O/O_2_ was administered during cannulation in 51% of ID sessions and 61% of DA sessions (NS, Fisher exact test). Oral or rectal midazolam premedication was administered for cannulation in 31% of ID sessions and 3% of DA sessions (p<0,001, Fisher exact test). Dental treatment was successful in 9 out of 10 sessions for both groups. Minor adverse effects occurred in 16.6% and 6.8% of ID and DA sessions respectively (p = 0.01, Fisher exact test). Patients with ID were more often very disturbed during cannulation (25.4% ID vs. 3.9% DA sessions) and were less often relaxed after induction (58.9% ID vs. 90.3% DA) and during dental treatment (39.5% ID vs. 59.7% DA) (p<0.001, Fisher exact test) than patients with DA. When midazolam sedation was repeated, cooperation improved for both groups. Conscious sedation procedures using intravenous midazolam, with or without premedication and/or inhalation sedation (50% N_2_O/O_2_), were shown to be safe and effective in patients with intellectual disability when administered by dentists.

## Introduction

A large minority of the population does not possess the cognitive capacity and adaptive skills required to cope with dental treatment and therefore cannot access therapeutic or preventive care. These difficulties particularly concern patients with cognitive impairment but other non-cognitively impaired patients, such as very young children or dentally anxious children and adults, also present behavioural difficulties during dental care leaving them vulnerable to poor oral health or undiagnosed problems [1]. For these patients, conscious sedation may improve the treatment experience whilst avoiding the use of physical restraint and thus may influence long-term levels of anxiety [2]. Numerous studies report the use of midazolam (Hypnovel®, Versed®), a short acting benzodiazepine, alone or with other drugs during dental treatment or other invasive outpatient care [3]–[6]. The UK definition of conscious sedation in dentistry is used in this article: “A technique in which the use of a drug or drugs produces a state of depression of the central nervous system enabling treatment to be carried out, but during which verbal contact with the patient is maintained throughout the period of sedation. The drugs and techniques used to provide conscious sedation for dental treatment should carry a margin of safety wide enough to render loss of consciousness unlikely” [7].

Published studies investigating the effectiveness and tolerance of midazolam used as a single agent for dental treatment are heterogeneous in their evaluation criteria, use different means of administration, concern different populations and are carried out in different settings [3], [4], [8]–[12]. In anxious adults without disability, the drug is usually administered intravenously. This route has many advantages. The presence of continuous venous access improves safety as it enables rapid injection of the antagonist flumazenil if necessary. Moreover, the intravenous route allows incremental titration of midazolam (usually 1 mg/min) and this limits the risk of respiratory depression [13]. Titration also reduces the risk of under- or over- sedation by allowing the clinician to obtain the desired level of sedation for each individual patient and for the type of treatment planned. Studies using intravenous midazolam in anxious patients have reported high success rates (evaluated by the percentage of planned dental treatment performed) without serious complication during oral surgery and dental treatment [3], [14], [15]. However, the level of cooperation or anxiety of the patient over the sessions has never been reported.

For young or anxious children, as for patients with intellectual disability, little specific data are available in these populations [16]–[22]. Midazolam is usually reported as being administered using an intranasal, intrarectal or oral route [23]–[27] because of potential better acceptability of these routes. Indeed, the acceptability of intravenous midazolam may be poor in patients that are often anxious about needles and failure of planned sedation may occur on cannulation in young children [28]. In a study comparing nitrous oxide to intravenous midazolam sedation for orthodontic extractions in adolescents, the main reason evoked for not participating in the trial was fear of cannulation (24% of 55 patients approached) [9]. However, the technique showed good effectiveness and safety when the IV midazolam protocol was accepted and IV midazolam was even preferred to nitrous oxide most of the time. One recent study also reported very good or excellent behaviour during dental treatment under IV sedation for 83% of 365 patients aged 7 to 16 years [29]. In adults with learning disability and challenging behaviour, two consecutive audits have investigated the use of intravenous titration to the sedation endpoint after intranasal midazolam premedication to facilitate cannulation [30], [31]. This technique allowed dental treatment to be carried out without major interference from the patient in 78.8% of cases [31]. Minor adverse events occurred in 6% of cases in these patients, of whom many had associated medical conditions. Unfortunately, neither the behaviour of the patients, nor the evolution of the psychological state of the patient over the session, were detailed.

The present study aimed to evaluate the effectiveness and safety of conscious sedation procedures using intravenous midazolam for dental care undertaken by trained hospital dentists in adults and children with intellectual disability (ID) compared with adults and children with dental anxiety (DA).

## Methods

### Ethics Statement

Three dental practitioners with postgraduate training in midazolam conscious sedation conducted this study at a University Hospital. All have ten to twenty years of experience of the routine use of conscious sedation using both 50% nitrous oxide/oxygen and midazolam IV, IR and PO, in populations with intellectual disability and/or anxiety. All are postgraduate teachers in conscious sedation at the Dental University and University Hospital of Clermont-Ferrand (France).

Approval was obtained from the local ethical comity (CCPPRB Auvergne i.e. “Comité Consultatif de Protection des Personnes se prêtant à la Recherche Biomédicale”) under registration number AU-571, and from the French National Drugs Agency (AFFSAPS), for an initial clinical trial entitled “Conscious sedation with midazolam in patients with dental anxiety: Impact of administration route (oral versus intravenous)”. The registration number on the “Clinical Trials” website for this study was NCT 01874717. However, oral administration was refused by patients or carers most of the time after IV administration or in some cases, an additional intravenous dose was necessary after oral administration. Consequently, in order to respect patients’ choice, the objectives of the study were changed and aimed at comparing patients with different cognitive profiles requiring IV sedation with midazolam for dental care. The Regional Clinical Research Division was immediately informed of this change and there was no additional requirement to resume the study. Furthermore, after update in the French ethical laws related to human research, global approval from the local ethical committee (n°CE-CIC-GREN-11/17) was obtained for all observational studies evaluating the quality of dental treatment performed in the dental university hospital of Clermont-Ferrand (France). This new text clearly stated that dental care under sedation or general anaesthesia was covered by this approval. The agreement specified that all patients visiting the unit should receive oral and written information mentioning that data obtained during dental care could be used anonymously for research purposes unless the patient specifically stated in writing otherwise (tacit agreement).

### Patients

All children and adults programmed for dental treatment under intravenous sedation with midazolam over a period of 50 months were considered for inclusion in the study. Exclusion criteria were: i) patients in the American Society of Anesthesiologists (ASA) category III, IV or V [32]; ii) patients having accepted dental treatment without premedication or sedation, and without declaring dental anxiety, during the month prior to the appointment; iii) Any medical contraindication to the use of midazolam. All patients were referred to the Unit of Special Care Dentistry by a general dental practitioner, a physician or member of the medical staff of a special institution. Conscious sedation with intravenous midazolam was planned if the patient could not be approached for more than a very brief examination, following failure to treat under inhalation sedation (50% N_2_O/O_2_), or following poor cooperation during treatment under inhalation sedation (50% N_2_O/O_2_). Poor cooperation was defined as a score of 3 or more on the French modified version of the Venham scale [33], [34] (Table 1).

**Table 1:** English translation of the French modified Venham Scale.

**0**	Relaxed, smiling, willing, able to converse, best possible working conditions; displays the behaviour desired by the dentist spontaneously, or immediately upon being asked
**1**	Uneasy, concerned; eye contact but tense facial expression; suspicious of environment; sits spontaneously back in the chair; hands remain down or partially raised to signal discomfort; during a stressful procedure may briefly and rapidly protest to demonstrate discomfort; the patient is willing and able to describe experience as requested; breath is sometimes held; capable of cooperating well with treatment.
**2**	Tense; tone of voice, questions and answers reflect anxiety; multiple requests for information; hands clench armrests or may be tense or raised without interfering with treatment; sits back spontaneously in chair but head and neck tense; accepts handholding; eye contact; during stressful procedure verbal protest, quiet crying; patient interprets situation with reasonable accuracy and continues to work to cope with his/her anxiety; protests more troublesome; patient still complies with request to cooperate; continuity is undisturbed
**3**	Reluctant; tends to reject the treatment situation, difficulty in assessing situational threat; frequent sighs; pronounced protest, crying; only sits back in chair after being asked several times, the head and neck remain tense; slight movements of avoidance; tense hands, avoids eye contact; accepts handholding; minor attempts to use hands to stop procedure; wriggling; protest out of proportion to threat or is expressed well before the threat; copes with situation with great reluctance; treatment proceeds with difficulty
**4**	Very disturbed by anxiety and unable to assess situation; physically very tense, wrinkled eyebrows, eye contact avoided or eyes shut; general crying not related to treatment; prominent avoiding movements, needing physical restraint on occasion; places hands over mouth or on dentist’s arm to prevent treatment, but eventually allows care to progress; pinches lips together but ends up by opening mouth; regularly lifts head from chair; rejects physical contact but may still accept handholding; patient can be reached through oral communication and eventually with reluctance and great effort begins to work to cope; dissociation is only partial; protest regularly disrupts procedure
**5**	Out of contact, fails to grasp the reality of the threat; inaccessible to oral and visual communication; rejects physical contact; clenches mouth and lips; closes mouth and clenches teeth whenever possible; violent head movements; screaming, shouting, swearing, fighting, aggressive; regardless of age, reverts to primitive flight responses; actively involved in escape behaviour; physical restraint required

Patients were assigned to two groups. Patients in the dental anxiety group (DA) expressed dental anxiety but had no intellectual disability. Patients in the intellectual disability group (ID) had an appropriate medical diagnosis or attended a special school, home or work placement. The children and adults in the ID group showed anxiety and/or poor cooperation due to difficulty interpreting the dental situation and due to functional and physical barriers to care.

A medical, dental and social evaluation of each patient was undertaken prior to intravenous sedation. This assessment confirmed the absence of a medical contraindication to the use of midazolam and revealed possible problems for intravenous cannulation (anatomical difficulties, difficulty coping with needles…). Patients were informed that an appropriate escort should be present from the start of the sedation session and remain with the patient over the following 24 hours. Oral and written pre- and post- operative instructions were given. When difficulties of anxiety or cooperation linked to the use of a needle were expressed by the patient or his/her carer, topical anaesthesia was prescribed (EMLA^®^ 5% cream: lidocaïne/prilocaïne 50/50) to be applied to the site of cannulation one hour before the appointment. Fasting was not required prior to intravenous sedation, but light meals only were recommended [35]. Treatment planning was also discussed with the patient and/or the carer, and consent to treat was obtained.

### Procedures

On attending for treatment under intravenous sedation, adherence to the preoperative instructions was verified and the escort was made fully aware of his or her postoperative role. Medical history and consent were confirmed. If necessary and possible, inhalation sedation (50% N_2_O/O_2_) was administered during cannulation in order to reduce anxiety and provide surface analgesia. When it was anticipated that inhalation sedation (50% N_2_O/O_2_) would be inadequate or impossible, an oral or rectal premedication was given. Commonly, oral midazolam was administered 10 to 20 minutes before cannulation (midazolam for injection at 5 mg/ml, (*Panpharma®)* mixed with sweetened syrup at the dose of 0.3 to 0.5 mg/kg). In certain patients, used to the administration of suppositories, intrarectal midazolam was occasionally administered (midazolam for injection at 5 mg/ml, (*Panpharma®)* given intra-rectally with a commercially available adapted syringe). In a few cases, oral hydroxyzine was prescribed 1½ hours before the appointment (Atarax®, 2 mg/kg). In one case, alprazolam was given to a patient with DA before implantology treatment (Xanax®, 0.25 mg the day before at bedtime and 0.25 mg one hour before dental treatment). If necessary, inhalation sedation (50% N_2_O/O_2_) was used in addition to premedication for cannulation but was removed during titration of midazolam in order to be able to gauge the sedative effect of the drug on behaviour and the physiological parameters. During dental treatment under IV midazolam, inhalation sedation (50% N_2_O/O_2_) was added if required in relation to the behaviour and to the physiological state of the patient. In particular, this technique was used to increase the level of sedation without further increasing the risk of respiratory depression. Oxygen was not systematically administered during the session.

The patient was monitored clinically from the moment of their arrival in the Unit. For most patients, a baseline physiological assessment was established before the administration of any drugs. For certain patients with marked opposition, sedation was commenced and the pulse oxymeter and blood pressure cuff placed as soon as physically possible. Systolic and diastolic blood pressure (SBP and DBP), heart rate (HR) and oxygen saturation (SpO_2_) were recorded throughout the sedation session and the recovery period (Monitor: DINAMAP ProCare 300). Following cannulation, intravenous midazolam (5 ml ampoules, 1 mg/ml, *Panpharma®*) was titrated (slow injection of 2 mg, wait 90 s, then titration in increments of 1 mg at 1 minute intervals) until a level of sedation was obtained that was sufficient for dental care to be performed in comfortable conditions for the patient.

From arrival at the hospital, and throughout the session and recovery period, behavioural management techniques were used continuously (e.g. maintenance of physical and verbal contact, positive reinforcement, reassurance, positive suggestion etc.). These techniques were adapted to the age and communication skills of the patient. Time was taken to introduce the patients to the different steps of the procedure and stress reducing strategies were used continually during care. Local anaesthesia was used systematically if there was the least risk of pain during treatment (including subgingival scaling or rubber dam clamp placement…).

### Study Criteria

1) The first objective was to study the effectiveness of the procedure. The three criteria used to assess effectiveness were the success of the treatment session, the level of cooperation during the session, and any positive change on repeat sedation.

- Success: The session was considered a ‘total success’ if the intended dental treatment was completed under intravenous sedation. It was a ‘partial success’ if only part of the planned dental care could be performed. ‘Failure’ was recorded if no dental treatment was possible. The type of treatment undertaken was also recorded.
- Level of cooperation: This was assessed using the French modified Venham scale [33], [34] (Table 1). This scale offers a good description of behaviour and anxiety in one score (from 0 and 5). The intra- and inter- examiner reliability of this scale has been previously confirmed [2]. Inter-investigator variability for the French modified Venham scale was controlled for the three dentists participating in the current study and was not statistically significant (General Linear Models procedure). The French modified Venham scale was applied at the following periods during the sedation session: Ti: At first contact with the dentist; T0: During venous cannulation; T1: At the end of the induction; T2: During the first injection of local anaesthesia; T3: At the moment of least cooperation during initial dental treatment.

Repeat sedation: The group of sessions corresponding to a first experience of intravenous sedation with midazolam was compared with the group of repeat sedation sessions. The success rate, the level of cooperation, the rate of adverse effects, and the dose of intravenous midazolam required were compared.

2) The second objective was to study the safety of the procedure. The criteria used to assess safety were: the incidence of adverse events during the sedation and recovery periods, the values of the recorded physiological parameters and the Ramsay score of level of sedation.

- Adverse events: These were pre-listed according to 5 categories: respiratory problems (hyper or hypoventilation, desaturation), digestive problems (nausea, vomiting), neurological problems (convulsions, epileptic fit…), behavioural events (euphoria, hyper-excitability…), vaso-vagal effects (sweating, pallor, faint…).
- Physiological parameters: The minimal level of oxygen saturation (SpO_2_), the minimal and maximal values of heart rate (HR), and systolic and diastolic blood pressure (SBP and DBP respectively) were recorded for each session. Normative values for physiological parameters were established (Table 2). The percentage of sessions with values outlying the normative range was analysed.
- Ramsay scores: The level of sedation was recorded applying Ramsay scale on first opening the mouth for examination after induction (T4), at the start of actual dental treatment (after local anaesthesia if necessary) (T5) and at the end of dental treatment (T6) [36] (Table 3). In order to meet the definition of conscious sedation, the Ramsay score should not exceed 3.

**Table 2:** Normative values of physiological parameters considered for this study.

	Heart Rate *(beats/min)*		Systolic Blood Pressure (mmHg)		Diastolic Blood Pressure (mmHg)	
Age (years)	Standards	*Normative range*	Standards	*Normative range*	Standards	*Normative range*
**11**	90	65< >160	95	70< >120	55	**40< >100**
**12–18**	70	60< >160	110	70< >130	58	**45< >110**
**Adult**	**75**	**50< >160**	**122**	**70< >145**	**75**	**50< >120**

**Table 3:** Ramsay sedation scale (Ramsay et al. 1974).

**1**	Anxious and agitated or restless, or both
**2**	Cooperative, oriented, and tranquil
**3**	Responsive to commands only
**4**	Exhibiting a brisk response to light glabellar tap or loud auditory stimulus
**5**	Exhibiting a sluggish response to light glabellar tap or loud auditory stimulus
**6**	Unresponsive

### Statistical Analysis

The statistical analysis was designed to study any potential differences between patients with DA and patients with ID as regards: age, gender, success rate, Venham scores, adverse events, Ramsay scores and percentage of sessions with physiological parameters out of the normal range. Statistical significance was set at *p*<0.05.

- Characteristics of the patients and progress of conscious sedation sessions: Age difference between the two groups was assessed by t-test. Gender distribution was compared by Fisher-exact test. Comparison of the mean duration of the conscious sedation sessions and of recovery time between the two groups, and comparison of the mean dose of intravenous midazolam, were undertaken using a t-test (α = 0.05). The frequency of the use of inhalation sedation (50% N_2_O/O_2_) and/or of premedication for cannulation, were compared between DA and ID groups using Fisher’s exact test.
- Success rate and adverse events: The Fisher’s exact test was used to compare the success rate and the percentage of adverse events between the two groups. The type of dental treatment performed was compared using the Pearson chi square test.
- Level of cooperation: The distribution of Venham scores at Ti, T0, T1, T2 and T3 was assessed for DA and ID groups. Three levels of cooperation were defined: i) Venham score 0: patient totally relaxed; ii) Venham score 1 to 3: moderate difficulties with cooperation; iii) Venham score 4 or 5: very disturbed patient, restraint necessary for treatment. Analysis of the distribution of the extreme levels (0 on one hand and 4–5 on the other) between patients with DA and with ID was undertaken using the Fisher exact test.
- Influence of repeat sessions: The group of sessions corresponding to a first experience of intravenous sedation with midazolam was compared with the group of repeat conscious sedation sessions. The success rate and the rate of adverse effects were compared with a Fisher exact test for DA and ID groups. The distribution of the extreme scores on the modified Venham scale (0 vs 4–5) at each time (Ti, T0, T1, T2, T3) was compared similarly. The dose of intravenous midazolam was also compared applying an independent t-test.
- Physiological parameters: Normative values for physiological parameters were established (Table 3). The percentage of sessions with SpO_2_, HR, SBP and DBP respectively outlying the normal range in the two groups was compared using the Fisher exact test.
- The distribution of Ramsay scores at T4, T5, and T6 was compared between groups using a Pearson chi square test.

## Results

### Characteristics of the Patients and Progress of Conscious Sedation Sessions

Ninety-eight patients with ID and 44 patients with DA were included in the study over 187 and 133 sessions of dental care under intravenous midazolam respectively. Demographic characteristics for DA and ID groups are given in Table 4. Both groups were similar in age (t-test) and gender (Fisher exact test). Thirty-eight sessions were performed in patients under 16 years. Thirty-three children with ID were included in the study with a mean age of 12.8±2.4 years (min: 8, max: 15). Five children with DA were included (7, 8, 13, 14 and 15 years old).

**Table 4:** Demographic characteristics of the DA (dental anxiety) and ID (intellectual disability) groups.

	No. Patients	No. Sessions	Male/Female n (%)	Age in years Mean (± SD) [min.-max.]	No. Sessions inpatients <16 years	Type of patients	Sessions(%)
**Patients with Dental Anxiety Disorder**	44	133	65/68 (48.9%/51.1%)	27.0 (±9.5) [7–66]	5	Associated medical condition (epilepsy, heart disorder, cancer, drug addiction)	5%
**Patients with** **Intellectual Disability**	98	187	113/74 (60.4%/39.6%)	30.5 (±11.6) [8–57]	29	Cerebral palsy	25.3%
						Autistic disorder	41.4%
						Learning difficulties	19.1%
						Down syndrome	13.6%
						Rare genetic syndrome	0.6%

The average duration of the sedation sessions from administration of premedication to the end of treatment was 72±28 min (min: 20, max: 160) and 103±36 min (min: 25, max: 258) for patients with ID and DA respectively (p<0.05, t-test). Inhalation sedation (50% N_2_O/O_2_) was administered during intravenous cannulation in 51.1% (94/184) of sessions for the ID group and 61.4% (81/132) of sessions for the DA group (NS, Fisher exact test). Inhalation sedation (50% N_2_O/O_2_) was administered during all or part of dental treatment in 50.0% (39/78) sessions performed in the ID group and 67.1% (47/70) sessions in the DA group. The mean dose of midazolam given intravenously was 8.8 mg ±4.9 mg (min: 3 mg, max: 27 mg) for the ID group and 9.8 mg ±4.1 mg (min: 2 mg, max: 28 mg) for the DA group (NS, t-test). The duration of titration of intravenous midazolam was correlated to the dose administrated (p<0.05, Pearson correlation). Premedication was given before cannulation in 58/187 (31%) sessions for patients with ID (midazolam: n = 55, hydroxyzine: n = 3) and in 4/133 (3%) sessions for patients with DA (oral midazolam: n = 2, hydroxyzine: n = 1, alprazolam: n = 1). Oral midazolam was the most common premedication for patients with ID (49/55). Rectal administration was used for 8 sessions in 8 different patients with ID. Recovery time was not different between the groups: 54±36 min (min: 10, max: 180) for patients with ID and 44±30 min (min: 0, max: 150) for patients with DA.

### Success Rate

Planned dental treatment was successfully performed in 90.6% of sessions in patients with DA and 89.1% in patients with ID, with no difference between groups (chi square test, NS). 7.1% of sessions in patients with DA and 10.3% of sessions in patients with ID were a ‘partial success’ (only part of the planned treatment completed). A total failure, or abandon of treatment, was recorded in 3 patients (2 with DA and 1 with ID), and these were subsequently referred for general anaesthesia. Of these patients, one 18 year old woman with DA received 0.5 mg/kg of oral midazolam and 8 mg of IV midazolam. She exhibited Venham scores of 5 throughout the session from the moment of cannulation. One 14 year old girl with DA received 6 mg of IV midazolam. She needed endodontic treatment for a first mandibular molar but she presented Venham scores of 3 or 4 throughout the session. One 13 year old boy with autistic disorder received 0.3 mg/kg of oral midazolam (but refused to drink all the preparation) and then 6 mg of IV midazolam. He also needed endodontic treatment for a first mandibular molar and exhibited scores of 3 or 4 throughout the session.

The type of dental treatment for which midazolam sedation was indicated was different between the groups (p<0,001, Pearson chi square test). More sessions for oral hygiene and scaling were undertaken in the group with ID and more conservative procedures (restorative or endodontic treatments) were performed for patients with DA (Table 5).

**Table 5:** Main dental treatment performed under intravenous midazolam sedation for patients with dental anxiety disorder (DA) and patients with intellectual disability (ID).

		Patients with DA	Patients with ID
		*No. sessions (%)*	*No. sessions (%)*
**Clinical assessment**	**Clinical examination and radiographs**	0 (0%)	1 (0.6%)
**Impression taking**		4 (3.3%)	9 (5.2%)
**Hygiene and periodontal treatment**	**Scaling without local anaesthesia**	0 (0%)	13 (7.5%)
	**Scaling with local anaesthesia**	1 (0.6%)	12 (6.9%)
**Conservative treatment**	**Restorative treatment without local anaesthesia**	0 (0%)	1 (0.6%)
	**Restorative treatment with local anaesthesia**	33 (27.5%)	33 (19%)
	**Root canal treatment with local anaesthesia**	37 (30.8%)	32 (18.4%)
**Prosthetics**	**Prosthetic treatment without local anaesthesia**	6 (5%)	13 (7.5%)
	**Prosthetic treatment with local anaesthesia**	9 (7.5%)	9 (5.2%)
**Oral surgery**	**Extraction (with local anaesthesia)**	24 (20%)	46 (26.4%)
	**Minor oral surgery (with local anaesthesia)**	6 (5%)	5 (2.9%)
	***Total no. sessions***	120	174

### Level of Cooperation

**Figure 1:**
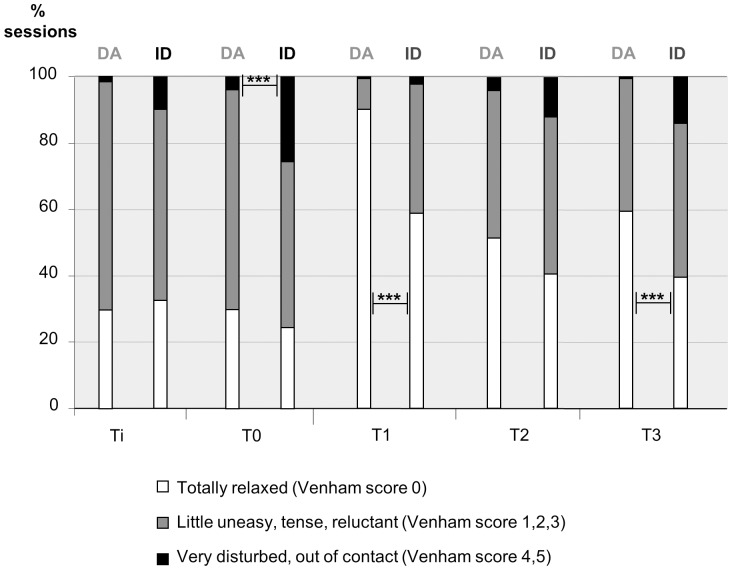
Comparison of cooperation scores between both groups during dental treatment. The distribution of the three levels of Venham score, at each time point and for each group. Ti: At first contact with the dentist; T0: During venous cannulation; T1: At the end of the induction; T2: During the first injection of local anaesthesia; T3: At the moment of least cooperation during initial dental treatment. DA: Group of patients with Dental anxiety disorder; ID: group of patients with Intellectual Disability. *** = significant difference between the two groups of patients (p<0.001, Fisher exact test).

The distribution of the three levels of Venham score, at each time point and for each group, is presented in Figure 1. When comparing only the extreme Venham scores (0 or 4–5), the following differences were demonstrated between patients with DA and those with ID: At T0, there were significantly more sessions with Venham scores of 4 or 5 for patients with ID (45/177, 25.4%) than for patients with DA (5/128, 3.9%) (p<0.001, Fisher exact test). At T1, significantly more sessions for patients with DA scored 0 than for patients with ID (112/124 (90.3%) vs. 99/168 (58.9%)) (p<0.001, Fisher exact test). At T3, in patients with ID, 70/177 (39.5%) sessions were undertaken with a totally relaxed patient (score 0), whereas in patients with DA 77/129 (59.7%) sessions were performed with a score of 0 (p<0.001, Fisher exact test). Statistical analysis of scores of 4 and 5 at Ti, T1, T2 and T3 was impossible because of the very low rates of these scores in patients with DA.

### Adverse Events

No major adverse event was recorded during the study. Minor side effects were recorded for 16.6% (31/187) of sessions in patients with ID and 6.8% (9/133) of sessions in patients with DA (p = 0.01, Fisher exact test) (Table 6). These rates were not influenced by concomitant psychotropic treatment when present (NS, Fisher exact test). The distribution of the type of adverse events was not different between groups. Minor adverse events were recorded in children under 16 years of age in 11 sessions, all in patients with ID (1 episode of desaturation, 2 of hiccups, 3 of nausea and 5 of hyperexcitability).

**Table 6:** Safety of the technique. Distribution of the different types of minor adverse events. DA = dental anxiety; ID = intellectual disability.

		DA Group		ID Group	
	*Total no. sessions:*	*133*	*type*	*187*	*type*
**Type of adverse events**	Respiratory (hyper- or hypo- ventilation; desaturation…)	1	1 apnoea (5 seconds)	8	8 desaturation lasting >30 seconds
	Neurological (convulsions, epilepsy…)	1	1 hiccups	8	6 hiccups,1 epileptic fit,1 hallucination
	Digestive (nausea, vomiting…)	6	3 vomiting (all in one womanwith severe gag reflex) and3 episodes of nausea	7	2 vomiting and 5 nausea
	Behavioural (euphoria, excitability…)	1	1 panic attack	8	6 hyperexcitability and 1 episode of lip-biting under local anaesthesia
	Vasovagal (sweating, pallor, faint…)	0		0	
	*Total no. events:*	*9 (6.8%)*		*31 (16.6%)*	

### Physiological Parameters

A print-out of physiological parameters was available for 110 sessions in patients with DA and 160 sessions in patients with ID. In 53 sessions, the poor level of cooperation of patients with ID did not allow baseline physiological assessment before initial induction with IV midazolam.

In 34 sessions the recorded SpO_2_ fell below 90%. Of these sessions, 8/110 (7.3%) were in patients with DA and 26/160 (16.3%) concerned patients with ID (p<0.05 Fisher exact test). In half of these sessions, low SpO_2_ was recorded in the induction period. In some cases, low recorded SpO_2_ was related to loss of the pulse oxymeter sensor from the finger. In all cases, low recorded SpO_2_ was corrected clinically by repositioning the sensor and by using simple airway management techniques (repositioning the patient to facilitate ventilation). Oxygen was administered (2l/min) in three sessions (for two patients with ID and one patient with DA), during 17 minutes in one case and 10 minutes in the others. Flumazenil was administered at the end of the session for one patient with intellectual disability in order to facilitate behaviour management during the return journey to his special home. It was never necessary to summon additional medical assistance.

In 17 sessions (12/110 in patients with DA and 5/160 in patients with ID), the HR fell below the minimal normative value in relation to the age of the patient (Table 3) (NS, Fisher Exact test). In 4 sessions (1/110 in patients with DA and 3/160 in patients with ID), it rose above over the maximal normative value (NS, Fisher Exact test).

The SBP was below the minimal normative value at some point for 3 sessions (all in patients with ID) and was above the maximal normative value at some point for 72 sessions: 17/110 (15.5%) in patients with DA and 55/160 (34.4%) in patients with ID (p<0.001, Fisher Exact test). The DBP was below the minimal normative value at some point for 27 sessions (11/110 in patients with DA and 16/160 in patients with ID) (Non Significant, Fisher Exact test) and was over the maximal normative value at some point for 12 sessions (all in patients with ID).

### Level of Sedation

The distribution of Ramsay scores at T4, T5 and T6 was different between patients with DA and patients with ID (p<0.001 at T4 and T6, and p<0.05 at T5, Pearson chi-square test) (Table 7). On opening the mouth for initial examination after induction (T4), the patients exhibited a Ramsay score of 1 to 3 in all cases and so did not exceed the limit of conscious sedation. At the start of actual dental treatment (T5), the Ramsay score was over 3 in 2 sessions in two patients with ID and 1 session in a patient with DA. At the end of treatment, when the stimulation of treatment was over, there were 4 sessions in the ID group with a Ramsay score of 4.

**Table 7:** Level of sedation. Distribution of the Ramsay scores recorded over the sessions (T4: on first opening of the mouth for examination after induction; T5: at the start of actual dental treatment; T6: at the end of dental treatment).

		Patients with Dental Anxiety	Patients with Intellectual Disability
Ramsay scale	Score	No. sessions (%)	No. sessions (%)
**T4**	*No. sessions*	*123*	*172*
	**1**	20 (16.3)	60 (34.9)
	**2**	96 (78)	83 (48,3)
	**3**	7 (5.7)	29 (16,9)
	**4**	0 (0)	0 (0)
	**5**	0 (0)	0 (0)
	**6**	0 (0)	0 (0)
**T5**	*No. sessions*	*121*	*167*
	**1**	15 (12.4)	43 (25.7)
	**2**	92 (76)	91 (54.5)
	**3**	13 (10.7)	31 (18.6)
	**4**	**1 (0.8)**	**1 (0.6)**
	**5**	0 (0)	**1 (0.6)**
	**6**	0 (0)	0(0)
**T6**	*No. sessions*	*122*	*169*
	**1**	11 (9.0)	52 (30.8)
	**2**	99 (81.1)	87 (51.5)
	**3**	12 (9.8)	26 (15,4)
	**4**	0 (0)	**4 (2.4)**
	**5**	0 (0)	0 (0)
	**6**	0 (0)	0 (0)

All patients subsequently recovered spontaneously without need for the use of flumazenil for reversal or additional medical assistance. However, all of these patients were contra-indicated for IV midazolam for future treatment as it was considered that the level of sedation necessary for treatment was over and above that of the self-imposed limit of conscious sedation [7].

### Influence of Repeat Conscious Sedation

**Figure 2:**
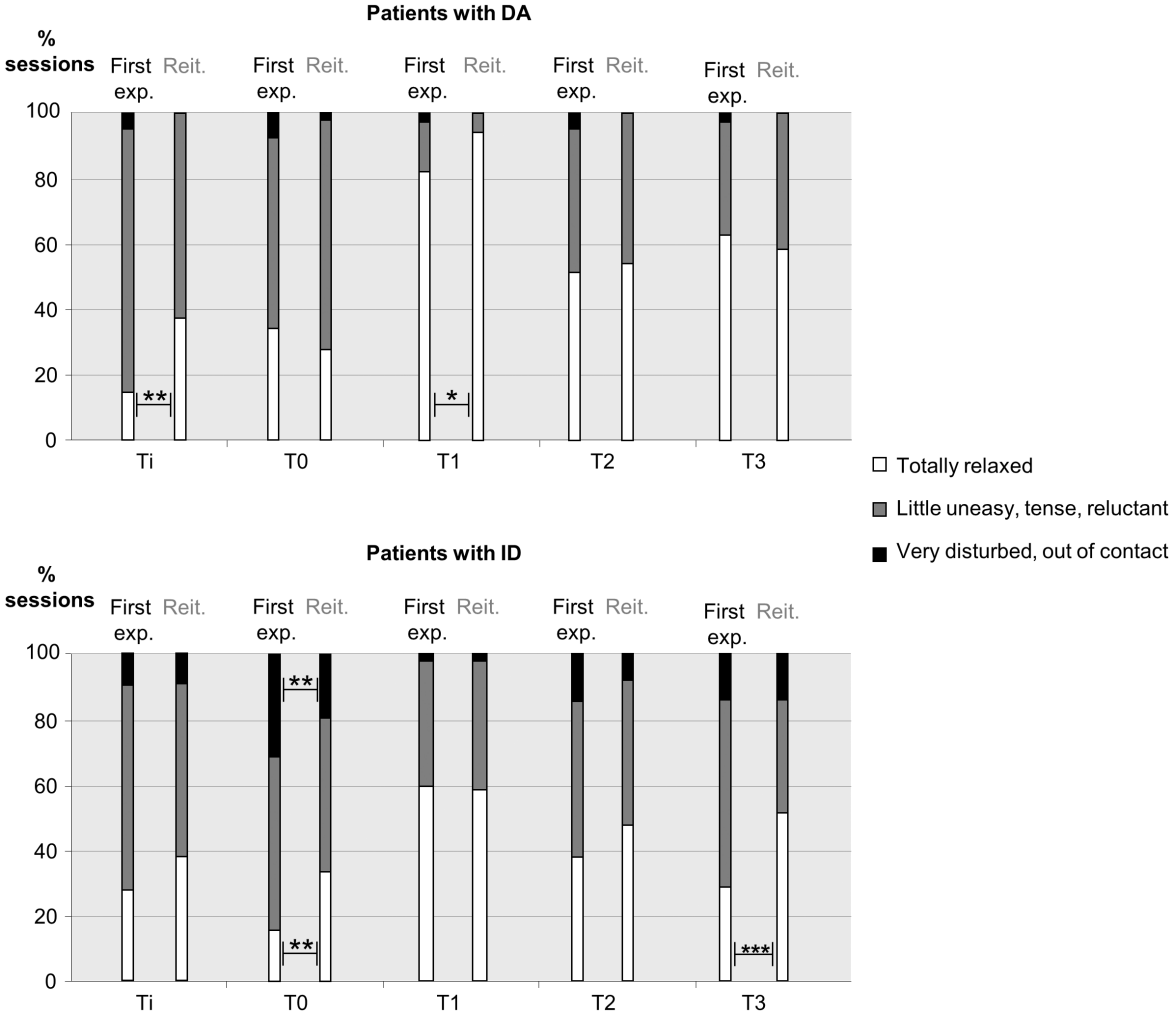
Influence of IV midazolam repetition on cooperation scores in both groups. The distribution of the three levels of Venham score, at each time point and for each group, in relation to the session being a first experience of sedation (First exp.) or a repeat session (Reit.). Ti: At first contact with the dentist; T0: During venous cannulation; T1: At the end of the induction; T2: During the first injection of local anaesthesia; T3: At the moment of least cooperation during initial dental treatment. DA: Group of patients with Dental Anxiety disorder; ID: group of patients with Intellectual Disability. Significant difference between the two groups of patients, Fisher exact test: *** = p<0.001, ** = p<0.01, * = p<0.05.

More sessions in the DA group were undertaken in patients with previous experience of the technique than for the group with ID (66.9% vs. 47.6%) (p<0.001, Pearson chi square test). The mean number of sessions per patient was 3.7±3.2 (min. 1, max. 16) for patients with DA and 2.3±2.4 (min. 1, max. 15) for patients with ID (p<0.001, t-test). When the sedation with intravenous midazolam was repeated, the rate of total and partial success combined increased significantly in patients with DA (from 92 to 99%, p<0.01 Fisher exact test) and remained statistically unchanged in patients with ID (from 96 to 99%). No influence of repetition on adverse events was found in either group. No influence of repetition on the dose of midazolam administered was found for either group (repeated measure procedure). For the repeated sessions in patients with DA, more sessions were conducted with a totally relaxed patient (Venham score of 0) at Ti (37.1% repetition vs. 14.6% first experience, p<0.01, Fisher exact test) and T1 (94.1% vs. 82.1%, p<0.05, Fisher exact test) (Figure 2). Statistical analysis was not possible for Venham scores of 4 or 5 because there were too few sessions with such scores. In patients with ID, more repeat sessions reported a Venham score of 0 at T0 (33.3% repetition vs. 15.6% first experience, p<0.01, Fisher exact test) and at T3 (51.2% vs. 28.6%, p<0.001, Fisher exact test). At T0, the patients were also less often very disturbed or out of contact (Venham scores of 4 or 5) when the procedure was repeated (19.5% vs. 31%, p<0.01, Fisher exact test). At T1, very few sessions were conducted with a Venham score of 4 or 5 so no statistical analysis was possible.

## Discussion and Conclusions

Conscious sedation procedures using intravenous midazolam administered with or without inhalation sedation (50% N_2_O_2_) or premedication were shown to be safe and effective in patients with intellectual disability as well as patients with dental anxiety disorder. This study is the first prospective clinical trial giving comparative data of conscious sedation for dental care in patients with intellectual disability and no influence of routine psychotropic treatment on the effectiveness or safety of the procedures used was found. In this study, thirty-eight sessions were performed in children and adolescents. More sessions would be necessary to allow a separate analysis of the young population.

The effectiveness of the conscious sedation could be partly explained by the flexibility of the approach used in order to address individual patient needs. In the vast majority of patients requiring premedication, the drug given was midazolam (per os or intrarectal) in order not to introduce multiple pharmacological effects (55/58 sessions). Inhalation sedation (50% N_2_O_2_) was used to allow cannulation and/or to deepen sedation without additional respiratory depression. The technique used was chosen in relation to each individual, in order to enable treatment with minimal distress at each step, from the beginning to the end of the care session. Effectiveness of the procedures also therefore depended on the ability of the operator/sedationist to evaluate and anticipate patient difficulties and behaviour prior to the session, according to the treatment required. The success rate of 89% showed that this procedure may be considered as an alternative to general anaesthesia for these patients, allowing comprehensive dental treatment and regular maintenance, as reported previously for this population [30], [31]. Three failures recorded during the study in two patients with DA and one patient with ID were related to very poor cooperation (Venham scores of 3 and more over the session, even with oral premedication) and to technical difficulties (molar endodontic treatment in young patients).Conscious sedation aims to reduce anxiety and improve cooperation for treatment. For this reason, the cooperation level was analysed by studying Venham scores in both groups. Patients with a dental anxiety disorder were significantly more often relaxed than patients with intellectual disability during actual dental treatment under intravenous midazolam sedation. Very few patients with dental anxiety exhibited very high Venham scores (4 or 5: very disturbed or out of contact). Similarly, a previous study showed that the sedative effect of 50% nitrous oxide in oxygen inhalation was more marked in patients with dental phobia compared with patients with intellectual disability [37]. These results suggest the difficulty in dealing with the very heterogeneous behavioural profiles present in the population of patients with intellectual disability [38]. Moreover, the average duration of sessions in this study was higher for anxious patients. It may be hypothesised that the relaxing psychological effect of the drug outlasts its pharmacological effect in patients without intellectual impairment. Subsequent cognitive restructuration was shown in anxious patients by their improvement in cooperation over repeat conscious sedation sessions before administration of the drug, even when anxiety was recorded on arrival in the surgery. When the protocol was repeated, patients with intellectual disability were also more often relaxed during the first steps of the session, in particular during cannulation. On repetition, fewer patients with ID had a Venham score of 4 or 5 (i.e fewer needed some type of physical restraint). During actual dental treatment, behaviour improved in patients with intellectual disability at repeat sessions and reached levels similar to that seen in persons with dental anxiety disorder. This confirmed the results of a previous study showing the educative and anxiolytic effects of repeat sessions of sedation by inhalation of 50% nitrous oxide in oxygen in patients with special needs [2]. The nature of this improvement needs to be determined by further investigations.

In over half of the sessions, inhalation sedation (50% N_2_O_2_) was used to reduce anxiety and nociception during cannulation, and then used throughout dental treatment if necessary. Previous studies conducted in children also showed that the association of nitrous oxide/oxygen with intravenous or oral midazolam enhances the effectiveness of the procedure [6], [28], [39]–[41]. Indeed, in one study, 80% of sessions were successfully conducted in cooperative patients responsive to verbal commands using a combination of nitrous oxide and midazolam versus 54% with midazolam alone [28]. The analgesic properties of nitrous oxide are particularly useful when combined with midazolam, especially during venous cannulation and injection of local anaesthesia. Moreover, nitrous oxide gives a supplemental sedative effect during treatment without respiratory depression. This is useful for those patients who respond more markedly to the hypnotic and depressive effects of midazolam than to its anxiolytic effects. Accordingly, some authors showed that when nitrous oxide was associated with midazolam for oral surgery there was a significant reduction in the amount of midazolam required, a significant reduction in recovery time, and a significant improvement in cooperation and arterial oxygen saturation [42].

In this study, the adequate dose of intravenous midazolam, not counting any premedication, was close to that reported in other recent articles [30], [31], and it was not different between groups. Some authors using midazolam plus propofol sedation found that higher doses were necessary for patients with intellectual disability [43]. However in the current study, premedication was given to 31% of patients with intellectual disability (midazolam in 94.8% of cases), to allow venous access or to allow placement of a mask for inhalation sedation. If these patients had been able to accept cannulation without premedication, it can be assumed that they would have required a greater quantity of IV midazolam to reach a suitable level of conscious sedation. Other authors have also reported that premedication is useful to increase acceptability of inhalation sedation [23]. The present study showed the safety of using inhalation sedation (50% N_2_O_2_) and/or premedication in association with intravenous midazolam sedation. This complements previous observations of the use of intranasal midazolam as premedication before cannulation in patients with intellectual disability [31]. The intranasal route of administration is not yet available in France but this technique is promising. It seems that the ability to associate different procedures is extremely important and allows for flexibility in relation to the needs of an individual patient and to the clinical context.

In the present study, as in that undertaken by Ransford et al. [31], the type of dental treatment performed was different between patients with a dental anxiety disorder and those with intellectual disability. This suggests that the main role of this procedure in the population with intellectual disability may be to enable simple regular maintenance and prevention and/or to enable examination for the most reluctant patients. For these patients, general anaesthesia may still be indicated if complex treatment is required. For patients with dental anxiety alone, intravenous midazolam sedation seems sufficient to be able to undertake all types of dental treatment.

No major adverse event occurred during the study and no relationship was found between the incidence of minor adverse events and concomitant psychotropic treatment when present. However, more minor adverse events occurred in patients with ID than in patients with DA (16.6% versus 6.8% respectively). In a previous study in adults with disability using intranasal plus intravenous midazolam, 6.0% of adverse events were reported [30]. In another study of children and adolescents during conscious sedation with oral or rectal midazolam, 15.4% of minor adverse effects were observed [25]. Other authors report a rate of 24.6% of minor adverse events during sedation with intranasal midazolam in children and adolescents [27]. The disparity between these results is in part due to differences in defining and recording minor adverse events.

Particular attentiveness is required for patients with disability as regards their physiological state during sedation. This study showed that the physiological parameters (minimal SpO_2_, maximal SBP and DBP) were outside of the normal range more often in these patients than in patients with dental anxiety disorder. The risk of serious complication is therefore higher in this population [44]. In particular, patients with motor difficulties or poor muscle tonicity (such as persons with Down syndrome) are at greater risk of respiratory depression than others. Moreover, the variations in blood pressure recorded here may suggest a higher level of stress during dental care in patients with intellectual disability despite sedation. This is related to greater problems with cooperation and the higher frequency of minor adverse events observed in this population [2]. Moreover, in this study, conscious sedation was surpassed on the Ramsay scale for 5 patients over 7 sessions (6 of which occurred in 4 patients with ID). The risk of over-sedation in this population should therefore not be underestimated. It is true that the inadequacy of the Ramsay scale for patients with communication deficiencies has been discussed by other authors, as verbal responsiveness cannot be considered a valid criterion to assess level of consciousness for these patients [32]. However, in the present study, the level of consciousness was evaluated according to the usual means of communication with the patient. Physiological parameters were monitored accurately during the period of loss of verbal contact and any variation managed appropriately. Although the boundary of conscious sedation was exceeded briefly in all cases, it was never necessary to reverse the sedation with flumazenil. All patients with ID and over-sedation were subsequently referred for general anaesthesia if further treatment was required. All these results concerning the safety of sedation suggest that particular attention should be paid in conscious sedation training to ensure that operator/sedationists are able to adapt to the behavioural, as well as physiological, particularities of each patient and to react adequately in case of over-sedation. Sufficient clinical training, ideally by mentoring, is required to ensure the effectiveness and safety of the procedure.
